# Normothermic Ex Vivo Liver Platform Using Porcine Slaughterhouse Livers for Disease Modeling

**DOI:** 10.3390/bioengineering9090471

**Published:** 2022-09-14

**Authors:** Melanie Krüger, Alicia Ruppelt, Benjamin Kappler, Elke Van Soest, Roos Anne Samsom, Guy C. M. Grinwis, Niels Geijsen, J. Bernd Helms, Marco Stijnen, Linda M. Kock, Marco Rasponi, Hans S. Kooistra, Bart Spee

**Affiliations:** 1 LifeTec Group BV, 5611 ZS Eindhoven, The Netherlands; 2 Department of Clinical Sciences, Faculty of Veterinary Medicine, Utrecht University, 3584 CT Utrecht, The Netherlands; 3 Dipartimento di Elettronica, Informazione e Bioingegneria, Politecnico di Milano, 20133 Milan, Italy; 4 Veterinary Pathology Diagnostic Centre, Department of Biomedical Health Sciences, Faculty of Veterinary Medicine, Utrecht University, 3508 TD Utrecht, The Netherlands; 5 Department of Biomolecular Health Sciences, Faculty of Veterinary Medicine, Utrecht University, 3584 CL Utrecht, The Netherlands; 6 Department of Biomedical Engineering, Eindhoven University of Technology, 5600 MB Eindhoven, The Netherlands

**Keywords:** hepatic diseases, machine perfusion, acetaminophen, steatosis

## Abstract

Metabolic and toxic liver disorders, such as fatty liver disease (steatosis) and drug-induced liver injury, are highly prevalent and potentially life-threatening. To allow for the study of these disorders from the early stages onward, without using experimental animals, we collected porcine livers in a slaughterhouse and perfused these livers normothermically. With our simplified protocol, the perfused slaughterhouse livers remained viable and functional over five hours of perfusion, as shown by hemodynamics, bile production, indocyanine green clearance, ammonia metabolism, gene expression and histology. As a proof-of-concept to study liver disorders, we show that an infusion of free fatty acids and acetaminophen results in early biochemical signs of liver damage, including reduced functionality. In conclusion, the present platform offers an accessible system to perform research in a functional, relevant large animal model while avoiding using experimental animals. With further improvements to the model, prolonged exposure could make this model a versatile tool for studying liver diseases and potential treatments.

## 1. Introduction

Liver diseases in humans impose a great burden on society, in terms of suffering and death as well as economically, and account for two million deaths per year worldwide [1,2,3]. More than 5500 liver transplants are performed in Europe each year, which currently is the only available treatment for end-stage liver diseases [4]. Moreover, there is a lack of understanding of the aetiology and/or pathogenesis for many of the most prevalent non-viral liver diseases, such as drug-induced liver injury (DILI) [5], alcoholic liver disease [6], liver cancer [7] and Metabolic Associated Fatty Liver Disease (MAFLD) [8].

Despite many efforts, neither the currently available in vivo nor in vitro systems represent satisfactory DILI models, in part due to the complexity of the diseases, and need to be improved significantly [9]. The latter also applies to the diagnosis and understanding of the hepatic diseases [10]. Therefore, there is a clear need for a reliable model to study liver diseases starting from very early stages to understand the underlying pathophysiology, design preventive strategies, improve diagnosis and find cures and treatments.

One option to study the effect of drugs, liver diseases and their treatments are ex vivo liver models. For many decades, organs have been kept viable outside the body through ex vivo machine perfusion. Recently, research evolved to preserve them for transplantation using ex vivo machine perfusion [11]. It keeps the organs in a physiological state without energy depletion and accumulation of waste products and, therefore, can also maintain organs with previous damage and extends utilization [11,12].

In transplantation research, different normothermic perfusion devices and protocols have been developed and applied to keep donor livers or donor liver grafts viable until transplantation [11,12,13,14,15,16,17,18,19,20,21,22]. Although these devices are mostly used for transplantation purposes, they can also be very valuable in a research setting [22], using for example, (slaughterhouse) animal livers. Most devices and methods are, however, very complex, which has led to reduced feasibility due to practical reasons, costs, and ease of use [22,23]. The design of the perfusion circuits varies widely in literature with regard to the number of pumps used, whether the system is draining openly or closed, and which oxygenators and heat exchangers are used [22]. Although all systems have a dual inflow into the hepatic artery (HA) and portal vein (PV), pressures and flows, the composition of the perfusion fluid as well as temperature, oxygen levels and perfusion duration differ among studies [22]. Generally, every pump introduces mechanical stress that can damage the red blood cells, although centrifugal pumps are overall less damaging than other pumps [24]. Therefore, it is more beneficial to use only one centrifugal pump if possible. In addition to a pump supplying the PV, a second pump is often used to enable a pulsatile inflow into the HA, although there is no clear significant advantage to pulsatility [22]. Many different additives are given to the perfusion fluids, such as anticoagulants, vasodilators, insulin, varying nutrients, antibiotics, or bile salts [22]. However, the need for many of these additives, except for anticoagulants, especially in short-term perfusion, is unclear. Some of them might even have adverse effects, such as insulin and glucose, that can cause glycogen depletion in hepatocytes [22]. Furthermore, the need for dialysis during short-term perfusion is unclear, as it is found to be not strictly necessary in the presence of sufficient nutrients [25].

Porcine organs are considered potential relevant models as they are of appropriate size for transplantation, have comparable anatomy, immunology [18] and bile composition [22] compared to humans. In a review by Maione et al. [22], porcine isolated liver perfusion models were assessed in terms of their usefulness for ischemic reperfusion injury research [22]. Unfortunately, most models had limitations, especially regarding reproducibility of results and standardization of models. Therefore, a model focused on the basic elementary components would assist in reaching this aim. All of the 16 reviewed models utilized pig livers harvested in animal experiments, to this date very few groups worked with slaughterhouse livers. The group of Grosse-Siestrup developed a perfused porcine model using livers obtained from a slaughterhouse [26], which was successfully utilized for demonstrating diclofenac toxicity [27] and fungal infections [28]. However, the maximum perfusion time was 220 min, which is probably due to increasing liver failure even in control livers as indicated by high levels of hepatic damage markers [26]. The circuit design contained three pumps, two of them roller pumps, and a dialysis unit [27,28], making the set-up not only complex but also increases the risk of hemolysis especially over extended perfusion periods. In another study by Izamis et al. [29], slaughterhouse porcine livers were perfused and examined with dynamic contrast-enhanced ultrasound, but could only be sustained on asanguineous perfusate for 3 h [29]. The group aimed to create a cost-effective and simple design, but mostly focused on the quality of perfusion and monitoring thereof—not the vitality and functionality of the livers. The use of another perfusion fluid than whole blood limits the usefulness in terms of hemodynamic parameters [22], viability and even cost-effectiveness of the model. Consequently, a simple and cost-effective design that is shown to preserve viability and functionality in a relevant ex vivo perfused liver model is lacking.

Although hepatoxicity has been tested previously on ex vivo slaughterhouse livers using diclofenac [27], currently no such model for acetaminophen toxicity exists. The only ex vivo liver models used for acetaminophen toxicity are based on human liver pieces [30], or animal experiments using pigs [31]. For steatosis only models introducing the disease in experimental animals exist [32], or alternatively, the much less representative precision-cut liver slices [33] or in vitro cell cultures [34].

We developed a minimal design and approach in order to be able to keep porcine livers obtained from a slaughterhouse viable outside of the body in a reproducible, stable manner. In this way, we intended to create a model that can be used for disease and treatment research instead of animal models, which is in compliance with the 3R principle without necessitating very complex and, therefore, costly and difficult aspects that could prevent such an application. To further assess the potential of the model for research purposes, we aimed to develop an ex vivo model representing the basic mechanisms that can lead to steatosis. Additionally, we aimed to verify whether hepatotoxicity can be studied in our model and, therefore, exposed the ex vivo liver to acetaminophen.

## 2. Materials and Methods

### 2.1. Liver and Blood Procurement

Livers were harvested from clinically healthy Dutch Landrace Hybrid pigs at four-to-six months of age and an average body weight of 110 kg. The pigs, raised for human consumption, were sedated with CO_2_ and subsequently exsanguinated at the slaughterhouse. The livers were collected after a warm ischemic time of 25 min after the death of the animal. Compliance of the slaughter process with the protocols of EC regulations 1069/2009 was supervised by the Dutch Ministry of Agriculture, Food and Food Quality and approved by the Food and Consumer Product Safety Authority. No ethical approval was required due to the fact that liver tissue was removed from already terminated animals. Animals were terminated for food production, but liver tissue is not used for human consumption. The complete gastrointestinal tract was excised from the pigs, and the liver was isolated from the tract subsequently. The bile duct was ligated immediately, and the portal vein was cannulated and subsequently flushed with 2 L of cold Custodiol^®^ solution (Dr. Franz Köhler Chemie GmbH, Bensheim, Germany) containing a total of 25,000 U heparin (5000 U/L, LEO Pharma, Amsterdam, The Netherlands). The perfusate was collected, stored and transported together with the liver at 4 °C.

Blood was pooled from two electro-stunned pigs per experiment in the slaughterhouse. After stunning, the pigs were hung from their hind legs and exsanguinated by opening the carotid arteries and jugular veins. On average, 7–8 L of blood was mixed with 550 mL of modified Tyrode’s solution to buffer the blood and maintain osmotic pressure, containing a total of 60,000 U heparin and stored and transported in a jerry can. The total time between the death of the pig, cold storage, and reperfusion was maximally 3.5 h. To minimize contamination during harvesting and blood collection, all instruments and transport components were packed and handled sterile.

### 2.2. Circuit Design

**Figure 1:**
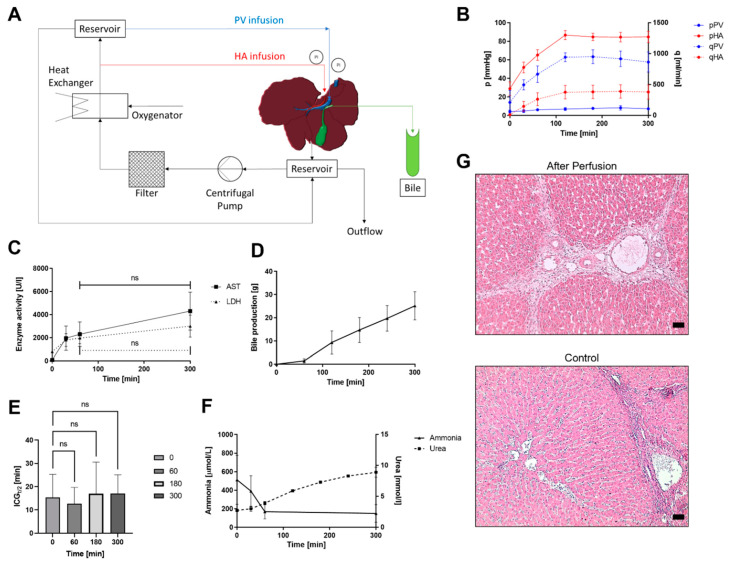
(**A**) Scheme of perfusion circuit design. (**B**) Portal vein and hepatic artery pressures (pPV and pHA) and flows (qPV and qHA) over 300 min of ex vivo liver perfusion with initially low starting pressures that reach physiological levels after 120 min. (**C**) Enzyme activity (LDH and AST) in blood over the perfusion duration with no statistically significant increase after the first 60 min. (**D**) Bile production over time, *n* = 3. (**E**) Indocyanine green (ICG) half-life of untreated livers measured at timepoints 0, 60, 180 and 300 without statistically significant differences. (**F**) Conversion of ammonia to urea over the perfusion period, scale bars represent 50 µm. (**G**) Representative H&E staining of control samples fresh from the slaughterhouse and after 5 h of perfusion with intact morphology; ns—not significant.

The liver was perfused through both the HA and PV with a pressure-driven continuous flow (Figure 1A and Figure S1). The perfusion fluid was circulated from the reservoir through an arterial filter (38 µm, Medtronic, Dublin, Ireland) and an oxygenator with an integrated heat exchanger (EOS ECMO, LivaNova, London, UK) into the liver and through the open vena cava back into the reservoir. The oxygenation level of the blood was maintained above 95%. The pressure in the HA was set by a centrifugal pump (Biomedicus 550, Dublin, Ireland), whereas the static pressure in the PV was achieved by controlling the height of a second reservoir. Pressures and flow rates were measured at the inlets of the HA and PV. The circuit was primed and deaired with 4.5 L of a modified Tyrode’s solution (priming solution), which was then supplemented with porcine blood to achieve a total perfusion volume of 7–8 L. The total perfusion volume was reduced to 5 L during the experiments with administration of continuous free fatty acid (FFA) and 300 mg/L acetaminophen (APAP).

### 2.3. Reperfusion

Prior to perfusion, the blood was filtered through a 200 µm filter (Sentinel filter, EATON, Dublin, Ireland) in order to remove any possible blood clots or contaminations. The blood was then oxygenated, and blood gas values were measured with a VetScan i-Stat 1 (Abaxis, Union City, CA, USA). Based on these values, the initial pH was set to a physiological level (7.35–7.45) using 1 mmol/L sodium bicarbonate, and the target of 1 mmol/L ionized calcium was balanced by using calcium chloride (both from VWR, Amsterdam, The Netherlands).

In the meantime, the liver was weighed and prepared on melting ice. The HA, bile duct and caudal vena cava were cannulated while ensuring that the liver vessels were free of air. Vena cava remained open to drain freely into a custom-built receptacle, and HA and PV were connected to the circuit while avoiding air from entering the vessels. To reduce reperfusion injury, reperfusion was started with a pressure of 22–35 mmHg in the HA and 3–5 mmHg in the PV. Blood rewarming was started upon reperfusion and set to 38 °C. As the resistance of the liver reduced over time, pressures were slowly increased for about an hour until they reached physiological levels of 90 (±9.5) mmHg in the HA and 7 (±2.3) mmHg in the PV [13].

After these hemodynamic values were reached, the livers were either perfused without further treatment or perfused with APAP or FFA. For livers without treatment (*n* = 3), bile production and blood gas parameters were measured every 60 min, pressures and flows were recorded, and pH, as well as the level of ionized calcium, were balanced. Blood samples were drawn at timepoints 0, 30, 60, 180, and 300 min and an indocyanine green (ICG) functionality assay was performed at timepoints 0, 60, 180 and 300 min. Tissue samples for histology were taken before and after 5 h of perfusion, and analysis was executed as described below.

### 2.4. Free Fatty Acid Treatment

To induce an early-stage steatotic liver, four perfused livers were treated with free fatty acids. Two perfused livers (FFA_bol) were treated with only one bolus mixture (t = 0) of 1.2 mM oleate (C18:1) (90% purity, Thermo Fisher GmbH, Kandel, Germany) and 0.6 mM palmitate (C16:0) (95% purity, Thermo Fisher GmbH) with 16% (*w*/*v*) fatty acid-free BSA (Fraction V, Gold Biotechnology, Inc., St. Louis, MO, USA) and two perfused livers (FFA_cts) were treated with the same initial bolus mixture (t = 0), but additionally, 25% of the initial dose was administered every 30 min to keep the concentration of free fatty acids in the liver at a constant level. The FFA bolus mixtures were administered with the start of reperfusion in order to allow the maximally available time for FFA metabolism. These concentrations were based on pilot experiments (data not shown) and literature [34,35]. During the experiment, bile production and blood gas parameters were measured every 60 min, pressures and flow rates were recorded, and pH, as well as ionized calcium, were balanced. Blood samples for hematology were taken and analyzed as described below. Additionally, tissue samples were flash frozen by first submerging them in isopropanol (2-propanol, Merck KGaA, Darmstadt, Germany) at −80 °C, then embedding them in Tissue-Tek optimal cutting temperature (O.C.T.) Compound (Sakura Finetek USA Inc., Torrance, CA, USA) and snap frozen in isopentane solution (VWR), samples were stored at −80 °C until further processing. Liver functionality was measured with an ICG assay, and the livers were weighed before and after perfusion.

### 2.5. Acetaminophen Treatment

To test acetaminophen toxicity on the ex vivo livers, 500 mg of the active component N-acetyl-para-aminophenol (APAP; Centrafarm Services B.V., Breda, The Netherlands) was dissolved in distilled water at a concentration of 4 mg/mL, and after dissolution, the NaCl level was adapted to the physiological level of 9 g/L. The APAP solution was administered with two different plasma concentrations. The first group received a bolus directly into the blood circulation (APAP_155, *n* = 3) to amount to 155 mg/L of APAP in plasma as it is between the toxic dose of 150 mg/L but below the lethal dose of 160 mg/L [36]. In the second group, a higher concentrated bolus was administered to amount to 300 mg/L plasma concentration (APAP_300, *n* = 3). Prior to APAP administration, the livers were perfused for 60–90 min to reach desired pressures and flows in order to avoid additional adverse effects on the liver during the initial reperfusion; the timepoint of APAP administration was defined as t = 0. Blood samples were taken 40 min before APAP administration and at the timepoints 10, 20, 30, 40, 60, 100, 140, 180, 220, and 300 min and treated and analyzed as described below. Blood gas measurements, pH, and ionized calcium level balance were executed at −40, 0, 60, 180, 220, and 300 min. Liver functionality was determined by an ICG assay, as described below. Tissue samples for histology were taken in the slaughterhouse, before the start of perfusion and after perfusion and were fixed in formalin as described below. The livers were also weighed before and after perfusion.

### 2.6. Analysis

Blood gas parameters were measured during the experiments, and pH and levels of ionized calcium were maintained manually. Bile production was equally measured by weighing the produced bile. Pressures and flow rates in HA and PV were monitored and recorded continuously. Blood samples were drawn from the oxygenator, centrifuged at 2300× *g* for 15 min and stored at −80 °C or 4 °C overnight, depending on the analysis. The next day, samples were transported to a clinical laboratory (Máxima Medisch Centrum, Veldhoven, The Netherlands) for analysis of concentrations of free fatty acid, urea, aspartate transaminase (AST), lactate dehydrogenase (LDH), ammonia, and lactate in a C8000 analyser (Roche Diagnostics International AG, Rotkreuz, Switzerland). After 5 h of perfusion, tissue samples were taken from an incision in the left lateral lobe with a biopsy punch (8 mm Ø, Megro GmbH & Co. KG, Wesel, Germany) for histology and RNA analysis. Tissue samples for histology were fixed in 10% *v*/*v* neutral-buffered formalin solution (4% *v*/*v* formaldehyde, Sigma-Aldrich, Zwijndrecht, The Netherlands) for one hour and then stored in 70% *v*/*v* ethanol (VWR) until further processing. Samples for RNA analysis were stored in RNAlater^®^ solution (Thermo Fisher Scientific, Eindhoven, The Netherlands) at −20 °C until further analysis. As a control, tissue samples from livers were taken directly after slaughtering in the slaughterhouse. After perfusion, the liver was weighed again.

### 2.7. Gene Expression

Biopsies stored in RNAlater^®^ (Thermo Fisher Scientific, Eindhoven, The Netherlands) were lysed with 350 µL RLT buffer (Qiagen, Hilden, Germany) containing 1% (*v*/*v*) 2-Mercaptoethanol (Sigma-Aldrich). Isolation of mRNA was done according to the manufacturer’s instructions with a RNeasy micro-kit (Qiagen), and the total mRNA was measured with a NanoDrop ND-1000 spectrophotometer (Thermo Fisher Scientific) at 260/280 nm. For the synthesis of complementary DNA (cDNA) from 500 ng mRNA, the manufacturer’s protocol for the used iScript cDNA Synthesis Kit (Bio-Rad, Hercules, CA, USA) was applied. Quantitative PCR (qPCR) was performed using a Bio-Rad CFX384 Real-Time PCR Detection System (Bio-Rad) with 10 ng cDNA per reaction and iQ™ SYBR^®^ Green Supermix (Bio-Rad). Primers are listed in Table S1. Data were analyzed with BioRad CFX manager software, and relative gene expression was calculated according to the 2^−ΔCT^ formula (E/100 × 2^−ΔCT^, with E = efficiency and ΔCT = mean threshold cycle gene of interest—mean reference gene threshold cycle) and normalized to the reference genes YWHAZ and RSP19. The normalized data was log-transformed for representation in the heatmap.

### 2.8. Histology

The formalin-fixed biopsies were routinely embedded in paraffin and cut into 5 µm sections before they were deparaffinized and rehydrated.

For immunohistochemistry, the slides were blocked in 10% (*v*/*v*) of normal goat serum (Sigma-Aldrich) in PBS after antigen retrieval. Antigen retrieval for albumin (ALB) and multi-drug resistance protein 2 (MRP2/ABCC2) staining was achieved in a citrate buffer (pH 6.0) for 30 min at 98 °C. For hepatocyte nuclear factor 4 alpha (HNF4a), antigen retrieval was performed in a Tris EDTA buffer (pH 9.0) for 30 min at 98 °C, and zonula occludens-1 (ZO1) antigen retrieval was performed with proteinase K (Dako, Santa Clara, CA, USA) for 10 min at room temperature (RT). The primary antibody for albumin (Sigma-Aldrich) was incubated at a 1:1000 dilution; HNF4a (LSbioscciences, Washington, DC, USA) in a 1:500 dilution; MRP2 (Monosan, Uden, The Netherlands) in a 1:1000 dilution; and ZO1 (Invitrogen, Carlsbad, CA, USA) in a 1:250 dilution, all incubations were performed overnight at 4 °C. The wash buffer for albumin, HNF4a, MRP2 and ZO1 was PBS with 0.1% (*v*/*v*) Tween 20. Envisioning of the antibodies was achieved with a Bright DAB substrate kit (VWR, PA, USA). As a counterstain hematoxylin (Dako, Glostrup, Denmark) was applied and slides were mounted with Vectamount (Vector Laboratories, Peterborough, United Kingdom) after dehydration. Images were taken on the Olympus BX60 microscope (Olympus, Leiderdorp, the Netherlands) and processed with Image-J software (NIH, Madison, WI, USA).

The H&E staining was performed according to a standard protocol. Incubation with hematoxylin was performed for 4 min at RT and eosin (Merck) was incubated for 1 min at RT.

For the Oil Red O staining, cryostat slides of 10 µm thickness were cut from fresh frozen liver tissues. The slides were fixed for 4 min with 4% (*w*/*v*) phosphate buffered formaldehyde (VWR) prior to incubation for 10 min with freshly prepared Oil Red O solution (VWR). Counterstaining was performed for 4 min with hematoxylin, and slides were mounted with Aqua-Mount medium (Thermo Fisher Scientific).

### 2.9. Indocyanine Green Functionality Assay

To perform a dynamic liver function test, indocyanine green (ICG, Carl Roth, 7695–2) was added at timepoints 0, 60, 180 and 300 min to the perfusate at 5 mg/L from a stock solution of 5 mg/mL. A blank sample was taken before ICG injection. After 2 min of circulation, the first sample was taken and set as timepoint zero (t = 0). Plasma samples (1 mL) were taken at 4, 6, 8, 10, 15, 20, 25 and 30 min after injection. Absorbance was measured at 805 nm with a CLARIOstar (BMG Labtech; Ortenberg, Germany) in triplicates. The ICG half-life was calculated as ln2/k from the fitted curve C_ICG_(t) = C_0_ × e ^–kt^. The ICG functionality test was only established as a standard analysis method for the untreated livers and the repetition of the experiments, which are livers treated with continuously administered free fatty acids and livers treated with 300 mg/L APAP.

### 2.10. Data Analysis

All data was processed, and statistical analysis where the sample size is n ≥ 3 was performed with GraphPad Prism 8 (San Diego, CA, USA). For the enzyme activity of untreated livers, an ordinary one-way ANOVA analysis was performed followed by a Tukey’s post-hoc test. Transaminase values for APAP-treated livers were compared using a one-tailed paired *t*-test. A comparison of liver functionality was performed using a two-way ANOVA followed by a Turkeys post-hoc test. All analyzed data was normally distributed with equal variances as tested by examining the Q-Q plot of residuals and scatter plots of residuals over predicted values. Differences were considered statistically significant for *p* < 0.05 and are represented by an asterisk, n refers to the sample size for each statistical analysis. Data are presented as mean values with standard deviation for all data sets.

## 3. Results

### 3.1. Untreated Livers

To determine the success of perfusion, hemodynamic parameters were assessed throughout the perfusion time. Reperfusion of the livers started with low pressures, which resulted in low flow rates through both the HA and PV (Figure 1B and Supplementary Table S2). At a starting pressure of 4.2 ± 1.3 mmHg in the PV, the initial flow was 211 ± 159 mL/min; whereas the pressure in the HA was 29 ± 6 mmHg, which resulted in a flow of 14 ± 11 mL/min. Upon rewarming and the reduction of the liver resistance, applied pressures were increased over time until 120 min when the physiological pressures were reached. The pressure of the PV of 7.2 ± 1 mmHg resulted in a flow of 864 ± 133 mL/min, and the HA pressure of 85 ± 5 mmHg led to perfusion of 380 ± 93 mL/min. Both flow rates remained constant during the perfusion time when pressures stayed on set values until termination of the experiment after 300 min.

To determine the viability of the perfused livers, the tissue injury markers LDH and AST were measured, and bile production was monitored as a sign of functionality. In Figure 1C, an initial increase of both enzymes is visible until 60 min of perfusion. LDH increased from 797 ± 15 U/L at the start of perfusion to 1967 ± 493 U/L at the end of perfusion, and AST increased from 89 ± 69 U/L to 2302 ± 1063 U/L. Over the subsequent 240 min, the LDH and AST levels did not increase significantly anymore. Figure 1D shows the bile production over time with a maximum total bile production of 25 ± 5 g with the first measurable production after 60 min of perfusion. H&E staining showed that the liver morphology after 5 h of perfusion was well maintained when compared to the control sample directly from the slaughterhouse (Figure 1G). The architecture of the liver was visible in both samples and the portal triads with PV. In addition, the HA and bile ducts were intact. After perfusion, the red blood cells were visible inside the tissue and mild sinusoidal dilatation was evident.

Liver function was further determined by ICG clearance from the perfusate, and an average half-life of 15.8 ± 8.4 min was determined for untreated livers during all measured timepoints without a significant increase in half-life over 5 h of perfusion (Figure 1E).

**Figure 2:**
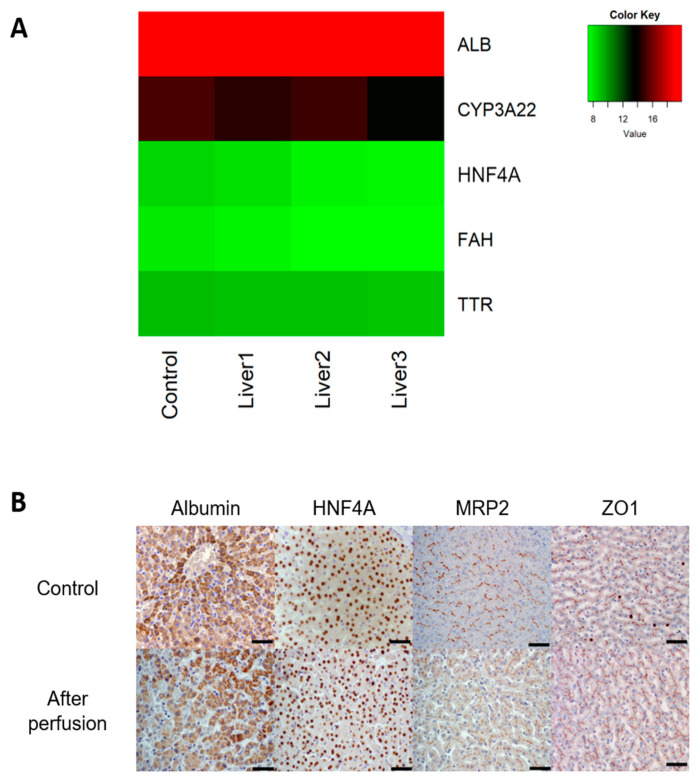
(**A**) Heatmap of DNA expression of ALB, CYP3A22, HNF4A, FAH, and TTR from liver samples taken directly in the slaughterhouse (control) and after 5 h of perfusion (Liver 1,2, and 3); DNA data is normalized to YWHAZ and RSP19 using the 2^−ΔCT^ formula and log-transformed, *n* = 3. (**B**) Representative immunohistology for control samples from the slaughterhouse and after 5 h of perfusion stained for albumin, HNF4A, MRP2, and ZO1. Scale bars represent 50 µm, *n* = 3.

Another indication of the functionality and metabolic activity of the liver is the conversion of ammonia to urea (Figure 1F). The initially high ammonia content in the blood of 745 ± 374 µmol/L reduced to 231 ± 71 µmol/L in the first 60 min, after which it remained at the same level. Urea production was first detected after 30 min, and it accumulated over time. Another liver function marker is glucose metabolism. Glucose levels showed an increase within the first 60 min of perfusion from 5.87 ± 0.83 mmol/L to 9.97 ± 3.41 mmol/L and decreased again to 5.53 ± 1.38 mmol/L after 5 h of perfusion (Table S2). To further evaluate the performance of the liver, gene expression levels of indicative liver proteins were measured (Figure 2A). Gene expression of the livers after perfusion was very similar compared to the control sample from the slaughterhouse for all tested samples. There was a high expression for the ALB gene encoding serum albumin as well as the cytochrome P450 isoform CYP3A22, the porcine analogue of human CYP3A4, a phase I specific enzyme. Hepatocyte nuclear factor 4 alpha (HNF4A), the genes encoding fumarylacetoacetate hydrolase (FAH), and transthyretin (TTR) were expressed at lower but equally constant levels.

For visualization of expression levels and localization within the liver acinus of albumin, HNF4A, MRP2, and ZO1, immunohistological stainings were performed (Figure 2B) in the perfused livers and compared to fresh livers directly from the slaughterhouse. Equal to the gene expression data, the overall expression levels were very similar between the control and perfused livers. Albumin staining was mainly found in the cytoplasm, some cells in the perfused liver did not show albumin expression, whereas all cells in the control were positive. HNF4A is a transcription factor in the nucleus of cells that should not stain outside the nuclei. In both perfused liver and the control HNF4A staining was specific in the nucleus and at the same level. Another marker, MRP2, is present in biliary transport and is expressed in hepatocytes at the canalicular/apical side. Especially in the perfused liver, MRP2 immunopositive staining was mainly seen in the area towards the bile canaliculi. Similarly, the tight junction protein ZO1 was located towards the bile canaliculi, and the expression levels were very similar for both the control and perfused samples.

### 3.2. Free Fatty Acid Treatment

**Figure 3:**
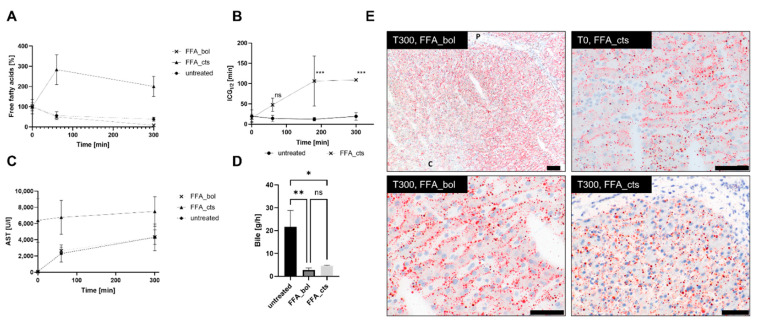
(**A**) Free fatty acid (FFA) concentration of untreated livers (*n* = 3), FFA_bol (*n* = 2) and FFA_cts (*n* = 2) over 5 h perfusion period. (**B**) ICG half-life of FFA_cts and untreated livers measure at timepoints 0, 60, 180 (*** *p* = 0.001) and 300 (*** *p* = 0.002). (**C**) Transaminase concentrations (AST) of FFA_bol, FFA_cts and untreated livers. (**D**) Total bile production after 5 h of perfusion, FFA_bol ** *p* = 0.0064, FFA_cts * *p* = 0.011. (**E**) Representative Oil Red O stainings of FFA_cts before treatment (T0) and of FFA_bol and FFA_ctst after 5 h of perfusion (T300). P—portal, C—centrilobular; scale bars represent 50 µm.

As proof of principle, two livers were treated with a high bolus concentrations of free fatty acids (FFA_bol) and two livers with a continued high concentration (FFA_cts) over 5 h of perfusion. The concentration of free fatty acids in the perfusate of FFA_bol was reduced by 93% at the end of perfusion, in the largest reduction took place within the first 60 min of perfusion (Figure 3A). The final FFA_bol concentration at the end of perfusion (0.215 ± 0.02 mmol/L) was comparable with the concentration found in the blood of untreated livers (0.154 ± 0.03 mmol/L). The free fatty acid concentration in the blood strongly increased in the first 60 min for FFA_cts by 180% and only slowly decreased insignificantly over the remaining 4 h of perfusion. In Figure 3E, Oil Red O staining visualizes the FFA in the tissue of FFA_bol and FFA_cts. The Oil Red O staining of FFA_bol at T300 showed the presence of numerous small and larger red globules in the tissue after treatment. The distribution appeared to be somewhat zonal, with a more prominent number of globules in the periportal area than in the centrilobular region. These globules seemed to follow the liver cell cords but often appeared to be located in the space of Disse or within the sinusoids and only infrequently within the hepatocytes. FFA_bol shows FFA droplets aligned in the trabecula at the beginning and end of perfusion, as well as FFA_cts at T0. However, this was not visible for FFA_cts at T300, where the FFA droplets did not align along clearly defined hepatic cords, indicating a disruption of the parenchymal architecture with dissociation of the hepatocytes. Additionally, only a limited presence of lipid droplets within the cytoplasm of hepatocytes was visible for both treatments. Notably, bile production was significantly reduced in the FFA-treated livers compared to untreated ones (2.8 ± 0.6 g/h and 4.6 ± 0.2 g/h for FFA_bol and FFA_cts, respectively, compared to 25 ± 5 g/h in untreated livers) (Figure 3D). The liver damage marker AST was not significantly increased during the 5 h of perfusion, but the livers with continuously administered FFA had higher AST levels compared to the untreated group, and those livers received only the bolus FFA. However, the AST levels in the FFA_cts perfusate were already increased prior to treatment (Figure 3C). Continuously administered FFA resulted in a clear increase of ICG half-life of 106 min ± 62 min after 3 h of perfusion compared to 12 min ± 4.3 min of untreated livers (Figure 3B). Also, the weight of the liver increased by 376 g compared to 164 g for untreated livers (Supplementary Table S3).

### 3.3. Acetaminophen Treatment

**Figure 4:**
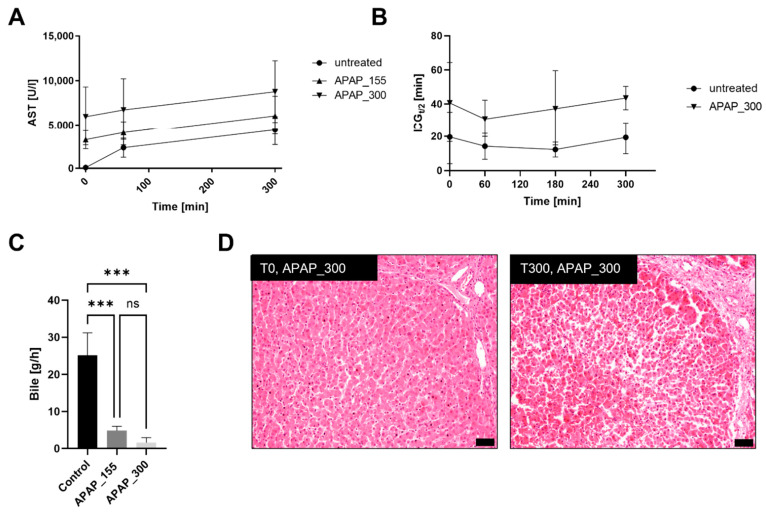
(**A**) Transaminase concentrations (AST) of acetaminophen (APAP)-treated livers (*n* = 3), APAP added at 0 min. (**B**) ICG half-life of APAP_300 and untreated livers measure at timepoints 0, 60, 180 and 300. (**C**) Bile production of APAP_155 (*** *p* = 0.0004), APAP_300 (*** *p* = 0.0001) and untreated livers. (**D**) Representative H&E staining of APAP_300 before the start of perfusion and after 300 min of APAP toxicity; scale bars represent 50 µm.

To assess the effect of APAP, transaminase released by the liver cells was measured in the blood. Average concentrations of AST did not increase significantly over 5 h of perfusion, but slightly higher AST plasma concentrations for 155 mg/mL (APAP_155) and 300 mg/mL (APAP_300) were measured compared to untreated livers (Figure 4A). H&E staining of APAP_300 samples showed a slight leukocyte infiltration before reperfusion, and prominent congestions were visible after 5 h of perfusion (Figure 4D). Some apoptosis or single cell necrosis were visible as well, but no zonal changes were seen in the hepatocytes. Furthermore, liver weight during the APAP perfusion, on average, increased by 421 g (see Supplementary Table S4), and glucose levels in blood decreased from 16.23 ± 13 mmol/L before APAP injection to 8.35 ± 5.8 mmol/L after 5 h of perfusion for APAP_155 and to 5.37 ± 0.61 mmol/L for APAP_300 (see Supplementary Table S4). Bile production was significantly reduced over 5 h of perfusion to 4.8 ± 0.9 g/h for APAP_155 and to 1.6 ± 1.1 g/h for APAP 300 compared to untreated livers (25 ± 5 g/h) (Figure 4C). Albumin production, on the other hand, was constant over time as measured for APAP_155 (see Supplementary Table S4). The ICG functionality test showed no significant increase in ICG half-life over 5 h of perfusion for APAP_300 (Figure 4B).

## 4. Discussion

We aimed to develop a straightforward design and protocol to perfuse slaughterhouse porcine livers in order to keep them viable and functional outside of the body in a stable and repeatable manner while complying with the 3R principle. This was successful, and perfusion periods were extended compared to previous studies using slaughterhouse organs [26,29]. Additionally, we were able to demonstrate the model’s potential to study the basic underlying mechanisms for liver diseases, such as a very novel ex vivo lipid uptake and to study the effects of compounds causing liver damage, such as acetaminophen.

Ex vivo porcine livers obtained from a slaughterhouse were perfused successfully in the laboratory. In vivo measurements of livers of pigs with slightly lower weight (90 kg) showed average PV flows of 1000 ± 200 mL/min and HA flows of 250 ± 70 mL/min [20], which shows that the final flows reached in our perfused slaughterhouse livers were at physiological levels without the need of added vasodilators as is common in ex vivo perfusion [20,22]. A limitation of using livers from a slaughterhouse where animals are used for human consumption is that the animal cannot be anticoagulated prior to harvesting or exsanguination. Hence, intrahepatic thrombosis is a major risk using slaughterhouse organs. Therefore, the livers were flushed directly after procurement with a heparinized flushing solution and transported submerged in a heparinized flushing solution to avoid intrahepatic thrombosis. Brüggenwirth et al. [37] described successful ex vivo perfusion for 24 h after a warm ischemic time of 30 min, also using slaughterhouse animals without injected anticoagulant prior to harvesting.

Our results showed restored flow to the desired values, and the histology of our control group showed well-preserved vessels. Therefore, we concluded that there is no severe intrahepatic thrombosis. In addition, using allogenic blood did not result in hemolytic reactions. This was also shown by Pool et al. [38], who compared the feasibility of porcine allogeneic blood in ex vivo normothermic perfusion with autologous blood. However, using autologous blood or cross-matching blood types might be necessary for long-term perfusion. Additionally, blood pooling can be avoided by reducing the perfusate volume needed in the circuit. This would also reduce the risk of contamination during blood collection. To minimize the number of additives during perfusion and during FFA and APAP administration, no antibiotic was used, which increased the risk of infections. However, during short-term (5 h) perfusion, the effect of infections is expected to be limited, and no pockets of infections were shown by histology. Nevertheless, to reach long-term perfusion, the addition of antibiotics is necessary [39], and future research should focus on the risk of bacterial infections due to slaughterhouse harvesting.

As a measure against reperfusion injury, which is a major issue in ex vivo but also clinical environments [22,40,41], we developed a protocol where starting pressures were low at first in order to allow adaptation of the livers and rewarm them slowly; the latter has been previously shown to have beneficial effects [18,42]. The former is hypothesized to be the reason why no vasodilators were needed in this set-up, as sinusoids are able to adapt to the conditions slowly with increasing pressures, which was seen when this protocol was not applied, as in these cases, physiological flows could not be reached (data not shown). This effect has also been reported in the literature, e.g., in coronary artery perfusion [43]. We also found that using our protocol, reperfusion injury indicated by an increase in AST as well as LDH in the first 60 min of perfusion and by histology was considerably less than when higher pressures were used at the start of reperfusion (data not shown). Levels are difficult to compare with literature data, as often liver weights are not reported, harvesting conditions or treatments are different, set-ups are different, and in all cases, more supplements were given to the blood [11,12,13,14,16,19,20,21,22,27,28,29].

In another study performed on slaughterhouse livers, AST levels reached on average 1695 ± 519 U/L after 90 min of perfusion [44] and LDH 1339 ± 249 U/L after 220 min [26], which is lower than that found after 60 min in the present study without significant changes over the remaining perfusion time (4304 ± 1347 U/L for AST and 3000 ± 778 U/L for LDH). One reason for the high AST levels could be a higher starting value due to a longer warm ischemic time, which is due to the particular slaughtering process. This time window was about 25 min in the present study; other procedures achieved 14 min [44] or less. It is well known that the duration of warm ischemic time corresponds to hepatic damage, specifically to hepatocytes, as expressed by increased AST levels [45]. This hypothesis is further supported by much lower damage marker values in experiments performed with lab animals [46]. The increase in both AST and LDH in combination with the observed dilated sinusoids during perfusion are a sign that liver cells are damaged during the change of physiological conditions of the ex vivo perfusion. Nevertheless, it can also be seen AST levels do not increase significantly after the initial rise; the remaining increase could limit function. Therefore, further improvements to the model should be aimed at stopping the damage marker increase while achieving longer perfusion times.

Longer-term studies have shown that recovery can occur in a previously damaged liver after only a few days [20]. Further experimentation with, for example, a slow increase in oxygen supply [18], or a short period of hypothermic oxygenated machine perfusion before normothermic perfusion [47], could aid in reducing initial damage in the future. Additionally, the effect of CO_2_ sedation as applied in the slaughterhouse used in this study on liver functionality needs to be further investigated to ensure no increase in initial damage in comparison to livers harvested from euthanized lab animals and ensure a consistent disease model using slaughterhouse livers.

As an indicator of the liver’s functionality [18], total bile production of around 25 g after 5 h with onset after 1 h of perfusion was measured, and when bile production is calculated over the last 4 h of perfusion with an average bile density of 928 kg/m3 [48], bile production averaged 6.7 mL/h. This bile production is comparable to data found in literature, 10 mL/h [13] after 5 h in non-slaughterhouse porcine livers, and 5.4 mL/h [45] after 90 min in slaughterhouse porcine livers. It has previously been shown that no significant depletion of bile salts occurs within the first 10 h of normothermic machine perfusion (NMP) [49], therefore its addition is unnecessary as proven by no reduction in bile production.

Another indicator of functionality is the synthesis of urea from ammonia [20]. Due to the closed circuit without a dialysis system, urea accumulation is expected and indicates metabolic activity in the hepatocytes [50]. This was 8.9 ± 0.67 mmol/L after 300 min in our study, comparable with literature data in slaughterhouse porcine livers of 10.3 ± 0.8 mmol/L of urea production after 220 min of perfusion [26]. The high urea production compared to ammonia elimination is a sign of the breakdown of proteins over the perfusion time, which indicates that the liver is in a catabolic state [51].

In addition, glucose metabolism in our group of untreated livers stabilized to blood glucose levels within the desired range (3.5–6.5 mmol/L), indicating liver functionality [20,52] without any addition of glucose or insulin within the 5 h of perfusion. However, a significant blood glucose increase was visible after 60 min of perfusion, which has been previously reported in the literature [37,52,53,54,55]. Becker et al. [55] described the release of glucose resulting in high blood glucose within the first phase after reperfusion as a consequence of ischemia-reperfusion injury and subsequent decrease of glucose levels, indicating the recovery of the liver. This was also shown by Izamis et al. [56], who described glucose release due to glycogenolysis within the first 2 h of perfusion after ischemic times.

Additionally, all gene expressions were stable over the time of perfusion, and especially the expression of albumin was constant at a high level, whereas albumin production usually decreases in the initial phase of NMP in most studies [20,32,45]. These findings were confirmed by the immunohistological stainings, which throughout demonstrated stable expression as well as localization of all markers. Furthermore, liver functionality was shown by ICG clearance from the perfusate. ICG is almost exclusively cleared by hepatocytes and excreted into bile without metabolism and enterohepatic recirculation. However, clearance also depends on liver blood flow and blood protein concentration [57,58].

Because stable blood flow and constant albumin expression were measured in the present study, the results of ICG clearance are clearly correlating to hepatocyte functionality and are not influenced by variations in blood protein concentrations or liver blood flow. The measured ICG half-life (15.8 ± 8.4 min) presents a comparable result to ICG half-life (approximately 14 min) in pigs with 30 min warm ischemia time, confirming the functionality of our ex vivo perfused livers [59]. The small increase in weight of the livers over the perfusion time in our study could be explained by the slight sinusoidal dilatation that was visible histologically but also indicated that no significant edema developed [60]. The exact nature of these lesions needed to be addressed in future studies. Therefore, we have shown that we can keep ex vivo porcine slaughterhouse livers in a viable and functional state. This was achieved with a less complex set-up compared to previous studies, which makes the application of the model for many research topics such as DILI, MAFLD, or other diseases more feasible due to the increased reproducibility and lower costs.

To demonstrate that the model has the potential to study the effects of diseases with respect to fat metabolism, such as steatosis, proof of principle experiments creating a damage model by supplying the livers with high FFA concentration were conducted. In the FFA bolus experiments, reduction of the FFA concentration within the first 60 min of perfusion showed that the liver takes up the fat and the Oil Red O staining confirmed this finding, although most of the lipids appeared to be located extracellularly. Extracellular localization of the droplets might be a temporal phenomenon, and hepatocytes eventually might take up the FFAs but require a longer perfusion than 300 min. Steatosis is known to start along the blood vessels [61], which was also observed in our study of the bolus FFA treatment. Early steatosis is characterized by lipid accumulation in macro- or microvesicular vacuoles in more than 5% of hepatocytes [62], which appeared to be in an early stage in this experiment as well. Observing that the majority of FFAs were taken up from perfusate by the liver in the first 60 min, FFA was administered continuously in a second experiment to keep the blood concentration high and to achieve an increase in severity. The continued supplementation of FFAs showed a rising concentration in the first 60 min of perfusion and only a slight decrease at a high level for the remaining 4 h of perfusion.

In addition, the ICG clearance showed a significant decrease in liver functionality compared to the untreated livers. ICG clearance has been reported previously as a successful measurement to identify already moderate steatotic livers [63], and therefore confirms early steatosis in our continuously treated model. Liver histology of the continuously supplied FFA after perfusion displayed a higher FFA concentration in the tissue compared to bolus and showed FFA present in the sinusoids as well as in the hepatocytes after perfusion, confirming the assumption of the liver taking up FFA. However, Oil Red O-stained liver sections visualized lipid accumulation already in tissue before reperfusion, indicating the presence of lipids without additional FFA administration in the harvested tissue. Further research needs to show if this could be a limitation of the model using slaughterhouse pigs due to the fact that pigs are fed to attain maximum growth, which might result in a higher blood FFA concentration. Nevertheless, continuous FFA administration resulted in increasing liver damage, as shown by the decreasing ICG clearance and reduced bile production. In the NMP perfusion of the already steatotic pig livers, the initial bile production was reduced before it recovered [32], showing that steatosis leads to decreased bile production, which is what is reflected in the much lower overall bile production in the FFA-treated livers compared to the untreated controls.

A further indication of liver damage is the increase in the weight of the liver during perfusion, which is commonly associated with hepatic steatosis [64]. Although both FFA administration protocols resulted in only limited lipid uptake into the hepatocytes without any visible necrosis or significantly increased damage markers, the results of our experiments suggest that the model is useful for studying early metabolic mechanisms and potential solutions to prevent the onset of a disease. With the prolongation of the exposure to FFA, the progression of the disease could potentially be studied, leading to insights into both the pathogenesis but also the potential of NMP for reducing steatosis [11,32]. Additionally, the timing for FFA administration could be improved with prolonged perfusion times.

In the present study, FFAs were administered from the start of reperfusion to exploit their possible uptake potential. The effect of FFA administration on reperfusion injury is unclear and starting the treatment after hemodynamic parameters have reached the target values might be beneficial to study the effects of FFAs on the liver. We have shown for the first time that it is possible in an ex vivo setting, utilizing slaughterhouse organs, to create a relevant metabolic model to study lipid uptake in livers which represents the first phase of the mechanism and, with further progression, could lead to a steatotic disease model. A larger sample size may lead to even more significantly relevant results. By learning about the kinetics and mechanism and attaining longer perfusion times, further disease stages might be achieved to create even more potential to study pathology.

In a further demonstration of an application of the perfused ex vivo porcine slaughterhouse liver model, a proof of principle study in the model was conducted with the well-known drug acetaminophen (APAP). Livers exposed to APAP do present only a few randomly distributed necrotic/apoptotic cells in the H&E stains, but the typical centrilobular lesions reported to be associated with APAP exposure [31,65] were not seen. However, a slightly higher concentration of the liver damage marker AST was measured after administration, which is also reported in the literature [66]. Nevertheless, the marker does not show as high increases as those found in vivo studies on pigs that progressed towards ALF [31,65]. In addition, ICG analysis did not show a decrease in functionality of livers treated with plasma concentrations of either 155 mg/mL or 300 mg/L. This is most likely due to the fact that only one bolus of APAP was given to show toxicity, whereas in most studied cases, plasma concentrations were kept at a constant level as opposed to the present study. Additionally, even higher concentrations of APAP could be needed to reach ALF in pigs [67,68]. Similar to other hepatoxicity studies using ex vivo perfused slaughterhouse pig livers [45], liver weight was increased in the current model over the time of perfusion, probably due to edema formation in livers.

A possible starting failure of the liver after APAP administration might be indicated by the observed reduction in blood glucose, as hypoglycemia is also associated with APAP intoxication [66,69]. The most striking difference could be found in bile production, which after APAP administration showed a significant decrease in total bile production after 5 h of perfusion compared to untreated livers. This might be a result of accumulating bile acids during APAP overdose, which also results in decreasing bile production [70,71]. Hence, it might be beneficial for future research to analyze the bile composition, which has been a promising biomarker for monitoring liver function [72].

When progressing the model to a further disease stage, additional parameters such as methemoglobinemia are interesting to investigate. An increase in liver damage and induced ALF could be achieved in an ex vivo NMP model without ethical implications for animal experiments when administering high doses of the drug over extended periods of time would be possible. Hence, to improve the potential of the current model for toxicity studies, we would further develop the model with continuous administration and for longer periods of time. Further progression could be of interest and extended research on continuous exposure and increased perfusion times are necessary to prove that the model can represent all stages of the disease and that it functions similarly to the in vivo models in all aspects.

## 5. Conclusions

We developed a simple ex vivo perfused liver model for research based on slaughterhouse material that avoids animal experimentation, is relevant in terms of its viability and functionality parameters without the burden of unnecessary complexity of the perfusion system. This has been successfully shown with parameters comparable to other experiments that even have more beneficial harvesting procedures like surgical environments or complex set-ups and protocols. Additionally, the model showed signs of liver damage due to acetaminophen and free fatty acid administration, which indicate a potential to use this setup to study basic metabolic mechanisms involved in the early stages of exposure to hepatotoxic substances and metabolic liver disease. With further improvements to the model, prolongation of exposure could lead to the progression of the disease, making this model a versatile tool for studying liver diseases and their potential treatments starting from the first phases.

## Data Availability

The research data that are not included in the article are available upon request from the corresponding author.

## Supplementary Materials

The following supporting information can be downloaded at: https://www.mdpi.com/article/10.3390/bioengineering9090471/s1, Figure S1: (**A**) Image of the custom-made liver receptacle with reservoir supplying the static portal vein pressure. (**B**) Cannulated porcine liver with gall bladder; Table S1: Primers used for DNA analysis; Table S2: Blood gas analysis, hematology, pressures and flows, bile production and liver weight for untreated at timepoints 0, 30, 60, 300 min of ex vivo perfusion as mean and SD over *n* = 3 livers (untreated); Table S3: Blood gas analysis, hematology, pressures and flows, bile production and liver weight for free fatty acid (FFA) treated livers at timepoints 0, 30, 60, and 300 min of ex vivo perfusion as mean and SD over *n* = 2 livers (FFA_bol, FFA_cts); Table S4: Hematology parameters and weight for livers treated with 155 mg/L and 300 mg/L acetaminophen (APAP) as mean and SD for *n* = 3 livers over 5 h of perfusion.

## References

[1] Gola A., Davis S., Greenslade L., Hopkins K., Low J., Marshall A., Thorburn D., Vickerstaff V., Jones L. (2015). Economic Analysis of Costs for Patients with End Stage Liver Disease over the Last Year of Life. BMJ Support. Palliat. Care.

[2] Pimpin L., Cortez-Pinto H., Negro F., Corbould E., Lazarus J.V., Webber L., Sheron N. (2018). Burden of Liver Disease in Europe: Epidemiology and Analysis of Risk Factors to Identify Prevention Policies. J. Hepatol..

[3] Asrani S.K., Devarbhavi H., Eaton J., Kamath P.S. (2019). Burden of Liver Diseases in the World. J. Hepatol..

[4] Blachier M., Leleu H., Peck-Radosavljevic M., Valla D.C., Roudot-Thoraval F. (2013). The Burden of Liver Disease in Europe: A Review of Available Epidemiological Data. J. Hepatol..

[5] Bale S.S., Vernetti L., Senutovitch N., Jindal R., Hegde M., Gough A., McCarty W.J., Bakan A., Bhushan A., Shun T.Y. (2014). In Vitro Platforms for Evaluating Liver Toxicity. Exp. Biol. Med..

[6] Kaplan W., Wirtz V.J., Mantel-Teeuwisse A., Stolk P., Duthey B., Laing R. (2013). Priority Medicines for Europe and the World 2013 Update Chapter 6.14. Harmful use of Alcohol, Alcohol Use Disorders and Alcoholic Liver Diseases. WHO Library Cataloguing-in-Publication Data Technical Report.

[7] Bruix J., Han K., Gores G., Llovet J.M., Mazzaferro V. (2015). Liver Cancer: Approaching a Personalized Care. J. Hepatol..

[8] Scherer A., Dufour J.F. (2016). Treatment of Non-Alcoholic Fatty Liver Disease. Dig. Dis..

[9] Kuna L., Bozic I., Kizivat T., Bojanic K., Mrso M., Kralj E., Smolic R., Wu G.Y., Smolic M. (2018). Models of Drug Induced Liver Injury (DILI)–Current Issues and Future Perspectives. Curr. Drug Metab..

[10] Kullak-Ublick G.A., Andrade R.J., Merz M., End P., Benesic A., Gerbes A.L., Aithal G.P. (2017). Drug-Induced Liver Injury: Recent Advances in Diagnosis and Risk Assessment. Gut.

[11] Weissenbacher A., Vrakas G., Nasralla D., Ceresa C.D.L. (2019). The Future of Organ Perfusion and Re-conditioning. Transpl. Int..

[12] Ravikumar R., Leuvenink H., Friend P.J. (2015). Normothermic Liver Preservation: A New Paradigm?. Transpl. Int..

[13] Butler A.J., Rees M.A., Wight D.G.D., Casey N.D., Alexander G., White D.J.G., Friend P.J. (2002). Successful Extracorporeal Porcine Liver Perfusion for 72 HR. Transplantation.

[14] Hessheimer A.J., Fondevila C., García-Valdecasas J.C. (2012). Extracorporeal Machine Liver Perfusion: Are We Warming Up?. Curr. Opin. Organ Transplant..

[15] Liu Q., Nassar A., Farias K., Buccini L., Mangino M.J., Baldwin W., Bennett A., O’Rourke C., Iuppa G., Soliman B.G. (2016). Comparing Normothermic Machine Perfusion Preservation with Different Perfusates on Porcine Livers from Donors after Circulatory Death. Am. J. Transplant..

[16] Nassar A., Liu Q., Farias K., D’Amico G., Tom C., Grady P., Bennett A., Diago Uso T., Eghtesad B., Kelly D. (2015). Ex Vivo Normothermic Machine Perfusion Is Safe, Simple, and Reliable: Results from a Large Animal Model. Surg. Innov..

[17] Jochmans I., Akhtar M.Z., Nasralla D., Kocabayoglu P., Boffa C., Kaisar M., Brat A., O’Callaghan J., Pengel L.H.M., Knight S. (2016). Past, Present, and Future of Dynamic Kidney and Liver Preservation and Resuscitation. Am. J. Transplant..

[18] Marecki H., Bozorgzadeh A., Porte R.J., Leuvenink H.G., Uygun K., Martins P.N. (2017). Liver Ex Situ Machine Perfusion Preservation: A Review of the Methodology and Results of Large Animal Studies and Clinical Trials. Liver Transplant..

[19] Ceresa C.D.L., Nasralla D., Jassem W. (2018). Normothermic Machine Preservation of the Liver: State of the Art. Curr. Transplant. Rep..

[20] Eshmuminov D., Becker D., Bautista Borrego L., Hefti M., Schuler M.J., Hagedorn C., Muller X., Mueller M., Onder C., Graf R. (2020). An Integrated Perfusion Machine Preserves Injured Human Livers for 1 Week. Nat. Biotechnol..

[21] Jayant K., Reccia I., Shapiro A.M.J. (2018). Normothermic Ex-Vivo Liver Perfusion: Where Do We Stand and Where to Reach?. Expert Rev. Gastroenterol. Hepatol..

[22] Maione F., Gilbo N., Lazzaro S., Friend P., Camussi G., Romagnoli R., Pirenne J., Jochmans I., Monbaliu D. (2018). Porcine Isolated Liver Perfusion for the Study of Ischemia Reperfusion Injury: A Systematic Review. Transplantation.

[23] Vogel T., Brockmann J.G., Friend P.J. (2010). Ex-Vivo Normothermic Liver Perfusion: An Update. Curr. Opin. Organ Transplant..

[24] Reddy S.P., Bhattacharjya S., Maniakin N., Greenwood J., Guerreiro D., Hughes D., Imber C.J., Pigott D.W., Fuggle S., Taylor R. (2004). Preservation of Porcine Non-Heart-Beating Donor Livers by Sequential Cold Storage and Warm Perfusion. Transplantation.

[25] Imber C.J., Peter S.D., De Cenarruzabeitia I.L., Pigott D., James T., Taylor R., Mcguire J., Butler A., Hughes D., Rees M. (2002). Advantages of normothermic perfusion over cold storage in liver preservation. Transplantation.

[26] Grosse-Siestrup C., Nagel S., Unger V., Meissler M., Pfeffer J., Fischer A., Groneberg D.A. (2001). The Isolated Perfused Liver: A New Model Using Autologous Blood and Porcine Slaughterhouse Organs. J. Pharmacol. Toxicol. Methods.

[27] Grosse-Siestrup C., Pfeffer J., Unger V., Nagel S., Witt C., Fischer A., David A. (2002). Isolated Hemoperfused Slaughterhouse Livers as a Valid Model to Study Hepatotoxicity. Toxicol. Pathol..

[28] Thewes S., Reed H.K., Grosse-Siestrup C., Groneberg D.A., Meissler M., Schaller M., Hube B. (2007). Haemoperfused Liver as an Ex Vivo Model for Organ Invasion of Candida Albicans. J. Med. Microbiol..

[29] Izamis M.L., Efstathiades A., Keravnou C., Leen E.L., Averkiou M.A. (2014). Dynamic Contrast-Enhanced Ultrasound of Slaughterhouse Porcine Livers in Machine Perfusion. Ultrasound Med. Biol..

[30] Schreiter T., Sowa J.P., Schlattjan M., Treckmann J., Paul A., Strucksberg K.H., Baba H.A., Odenthal M., Gieseler R.K., Gerken G. (2016). Human Ex-Vivo Liver Model for Acetaminophen-Induced Liver Damage. Sci. Rep..

[31] Newsome P.N., Henderson N.C., Nelson L.J., Dabos C., Filippi C., Bellamy C., Howie F., Clutton R.E., Lee A., King T. (2010). Development of an Invasively Monitored Porcine Model of Acetaminophen-Induced Acute Liver Failure. BMC Gastroenterol..

[32] Jamieson R.W., Zilvetti M., Roy D., Hughes D., Morovat A., Coussios C.C., Friend P.J. (2011). Hepatic Steatosis and Normothermic Perfusion—Preliminary Experiments in a Porcine Model. Transplantation.

[33] Westra I.M. (2014). Precision-Cut Liver Slices: An Ex Vivo Model for the Early Onset and End-Stage of Liver Fibrosis.

[34] Van der Laan L.J.W., Geijsen N., Spee B., Huch M., Schotanus B.A., Verstegen M.M.A., Grinwis G.C.M., Vernooij I.G.W.H., Oosterhoff L.A., Roesch C. (2017). Long-Term Adult Feline Liver Organoid Cultures for Disease Modeling of Hepatic Steatosis. Stem Cell Rep..

[35] Gómez-Lechón M.J., Donato M.T., Martínez-Romero A., Jiménez N., Castell J.V., O’Connor J.E. (2007). A Human Hepatocellular in Vitro Model to Investigate Steatosis. Chem. Biol. Interact..

[36] Winek C.L., Wahba W.W., Winek C.L., Balzer T.W. (2001). Drug and Chemical Blood-Level Data 2001. Forensic Sci. Int..

[37] Brüggenwirth I.M.A., van Leeuwen O.B., de Vries Y., Bodewes S.B., Adelmeijer J., Wiersema-Buist J., Lisman T., Martins P.N., de Meijer V.E., Porte R.J. (2020). Extended Hypothermic Oxygenated Machine Perfusion Enables Ex Situ Preservation of Porcine Livers for up to 24 Hours. JHEP Rep..

[38] Pool M.B.F., Hartveld L., Leuvenink H.G.D., Moers C. (2020). Normothermic Machine Perfusion of Ischaemically Damaged Porcine Kidneys with Autologous, Allogeneic Porcine and Human Red Blood Cells. PLoS ONE.

[39] Eshmuminov D., Mueller M., Brugger S.D., Bautista Borrego L., Becker D., Hefti M., Hagedorn C., Duskabilova M., Tibbitt M.W., Dutkowski P. (2021). Sources and Prevention of Graft Infection during Long-Term Ex Situ Liver Perfusion. Transpl. Infect. Dis..

[40] Mendes-Braz M., Elias-Miró M., Jiménez-Castro M.B., Casillas-Ramírez A., Ramalho F.S., Peralta C. (2012). The Current State of Knowledge of Hepatic Ischemia-Reperfusion Injury Based on Its Study in Experimental Models. J. Biomed. Biotechnol..

[41] Siniscalchi A. (2016). Post Reperfusion Syndrome during Liver Transplantation: From Pathophysiology to Therapy and Preventive Strategies. World J. Gastroenterol..

[42] Banan B., Xiao Z., Watson R., Xu M., Jia J., Upadhya G.A., Mohanakumar T., Lin Y., Chapman W. (2016). Novel Strategy to Decrease Reperfusion Injuries and Improve Function of Cold-Preserved Livers Using Normothermic Ex Vivo Liver Perfusion Machine. Liver Transplant..

[43] Fujita S., Roerig D.L., Bosnjak Z.J., Stowe D.F. (1998). Effects of Vasodilators and Perfusion Pressure on Coronary Flow and Simultaneous Release of Nitric Oxide from Guinea Pig Isolated Hearts. Cardiovasc. Res..

[44] Grosse-Siestrup C., Unger V., Pfeffer J., Dinh Q.T., Nagel S., Springer J., Witt C., Wussow A., Groneberg D.A. (2004). Hepatotoxic Effects of Polidocanol in a Model of Autologously Perfused Porcine Livers. Arch. Toxicol..

[45] Schön M.R., Kollmar O., Akkoc N., Matthes M., Wolf S., Schrem H., Tominaga M., Keech G., Neuhaus P. (1998). Cold Ischemia Affects Sinusoidal Endothelial Cells While Warm Ischemia Affects Hepatocytes in Liver Transplantation. Transplant. Proc..

[46] Gilbo N., Wylin T., Heedfeld V., Jochmans I., Pirenne J., Friend P., Monbaliu D. (2022). Porcine Liver Normothermic Machine Perfusion: Methodological Framework and Potential Pitfalls. Transplant. Direct.

[47] Boteon Y.L., Laing R.W., Schlegel A., Wallace L., Smith A., Attard J., Bhogal R.H., Neil D.A.H., Hübscher S., Thamara M. (2018). Combined Hypothermic and Normothermic Machine Perfusion Improves Functional Recovery of Extended Criteria Donor Livers. Liver Transplant..

[48] McIntosh R.L., Anderson V. (2010). A Comprehensive Tissue Properties Database Provided for the Thermal Assessment of a Human at Rest. Biophys. Rev. Lett..

[49] Eshmuminov D., Leoni F., Schneider M.A., Becker D., Muller X., Onder C., Hefti M., Schuler M.J., Dutkowski P., Graf R. (2018). Perfusion Settings and Additives in Liver Normothermic Machine Perfusion with Red Blood Cells as Oxygen Carrier. A Systematic Review of Human and Porcine Perfusion Protocols. Transpl. Int..

[50] Reiling J., Lockwood D.S.R., Simpson A.H., Campbell C.M., Bridle K.R., Santrampurwala N., Britton L.J., Crawford D.H.G., Dejong C.H.C., Fawcett J. (2015). Urea Production during Normothermic Machine Perfusion: Price of Success?. Liver Transplant..

[51] Bruinsma B.G., Sridharan G.V., Weeder P.D., Avruch J.H., Saeidi N., Özer S., Geerts S., Porte R.J., Heger M., Van Gulik T.M. (2016). Metabolic Profiling during Ex Vivo Machine Perfusion of the Human Liver. Sci. Rep..

[52] Sutton M.E., Op Den Dries S., Karimian N., Weeder P.D., De Boer M.T., Wiersema-Buist J., Gouw A.S.H., Leuvenink H.G.D., Lisman T., Porte R.J. (2014). Criteria for Viability Assessment of Discarded Human Donor Livers during Ex Vivo Normothermic Machine Perfusion. PLoS ONE.

[53] Matton A.P.M., De Vries Y., Burlage L.C., Van Rijn R., Fujiyoshi M., De Meijer V.E., De Boer M.T., De Kleine R.H.J., Verkade H.J., Gouw A.S.H. (2019). Biliary Bicarbonate, PH, and Glucose Are Suitable Biomarkers of Biliary Viability During Ex Situ Normothermic Machine Perfusion of Human Donor Livers. Transplantation.

[54] Matton A.P.M., Burlage L.C., van Rijn R., de Vries Y., Karangwa S.A., Nijsten M.W., Gouw A.S.H., Wiersema-Buist J., Adelmeijer J., Westerkamp A.C. (2018). Normothermic Machine Perfusion of Donor Livers without the Need for Human Blood Products. Liver Transplant..

[55] Becker D., Eshmuminov D., Keller R., Mueller M., Bautista Borrego L., Hagedorn C., Duskabilova M., Tibbitt M.W., Onder C., Clavien P.A. (2021). Automated Insulin Delivery-Continuous Blood Glucose Control during Ex Situ Liver Perfusion. IEEE Trans. Biomed. Eng..

[56] Izamis M.L., Tolboom H., Uygun B., Berthiaume F., Yarmush M.L., Uygun K. (2013). Resuscitation of Ischemic Donor Livers with Normothermic Machine Perfusion: A Metabolic Flux Analysis of Treatment in Rats. PLoS ONE.

[57] Ott P., Keiding S., Bass L. (1992). Intrinsic Hepatic Clearance of Indocyanine Green in the Pig: Dependence on Plasma Protein Concentration. Eur. J. Clin. Investig..

[58] De Gasperi A., Mazza E., Prosperi M. (2016). Indocyanine Green Kinetics to Assess Liver Function: Ready for a Clinical Dynamic Assessment in Major Liver Surgery?. World J. Hepatol..

[59] Lamesch P., Raygrotzki S., Evers B., Pichlmayr R. (1992). ICG Test Als Verlaufsparameter Nach Unterschiedlichen Ischämiebelastungen Der Leber–Eine Tierexperimentelle Studie. Chirurgisches Forum ’92 Für Experimentelle und Klinische Forschung.

[60] Imber C.J., Peter S.D.S., De Cenarruzabeitia I.L., Lemonde H., Rees M., Butler A., Clayton P.T., Friend P.J. (2002). Optimisation of Bile Production during Normothermic Preservation of Porcine Livers. Am. J. Transplant..

[61] Schleicher J., Dahmen U., Guthke R., Schuster S. (2017). Zonation of Hepatic Fat Accumulation: Insights from Mathematical Modelling of Nutrient Gradients and Fatty Acid Uptake. J. R. Soc. Interface.

[62] Kanuri G., Bergheim I. (2013). In Vitro and in Vivo Models of Non-Alcoholic Fatty Liver Disease (NAFLD). Int. J. Mol. Sci..

[63] Seifalian A.M., El-Desoky A., Davidson B.R. (2001). Hepatic Indocyanine Green Uptake and Excretion in a Rabbit Model of Steatosis. Eur. Surg. Res..

[64] Nassir F., Rector R.S., Hammoud G.M., Ibdah J.A. (2015). Pathogenesis and Prevention of Hepatic Steatosis. Gastroenterol. Hepatol..

[65] Thiel C., Thiel K., Etspueler A., Morgalla M.H., Rubitschek S., Schmid S., Steurer W., Königsrainer A., Schenk M. (2011). A Reproducible Porcine Model of Acute Liver Failure Induced by Intrajejunal Acetaminophen Administration. Eur. Surg. Res..

[66] Manibur Rahman T., Humphrey J.F. (2000). Hodgson Animal Models of Acute Hepatic Failure. Int. J. Exp. Pathol..

[67] He G.-L., Feng L., Cai L., Zhou C.-J., Cheng Y., Jiang Z.-S., Pan M.-X., Gao Y. (2017). Artificial Liver Support in Pigs with Acetaminophen-Induced Acute Liver Failure. World J. Gastroenterol..

[68] Dargue R., Zia R., Lau C., Nicholls A.W., Dare T.O., Lee K., Jalan R., Coen M., Wilson I.D. (2020). Metabolism and Effects on Endogenous Metabolism of Paracetamol (Acetaminophen) in a Porcine Model of Liver Failure. Toxicol. Sci..

[69] Beltrán-Olazábal A., Martínez-Galán P., Castejón-Moreno R., García-Moreno M.E., García-Muro C., Esteban-Zubero E. (2019). Management of Acetaminophen Toxicity, a Review. Iberoam. J. Med..

[70] Nasralla D., Coussios C.C., Mergental H., Akhtar M.Z., Butler A.J., Ceresa C.D.L., Chiocchia V., Dutton S.J., García-Valdecasas J.C., Heaton N. (2018). A Randomized Trial of Normothermic Preservation in Liver Transplantation. Nature.

[71] Ghallab A., Hassan R., Hofmann U., Friebel A., Hobloss Z., Brackhagen L., Begher-Tibbe B., Myllys M., Reinders J., Overbeck N. (2022). Interruption of Bile Acid Uptake by Hepatocytes after Acetaminophen Overdose Ameliorates Hepatotoxicity.

[72] Brüggenwirth I.M.A., Porte R.J., Martins P.N. (2020). Bile Composition as a Diagnostic and Prognostic Tool in Liver Transplantation. Liver Transplant..

