# Supplementary material for: Normothermic Ex Vivo Liver Platform Using Porcine Slaughterhouse Livers for Disease Modeling
Source: Bioengineering (Basel). 2022 Sep 14;9(9):471. doi: 10.3390/bioengineering9090471 (PMC9495507; doi:10.3390/bioengineering9090471)
Supplement: Supplementary file 1 [file bioengineering-09-00471-s001.zip › bioengineering-1851601-supplementary.pdf]

bioengineering

Supplementary Materials

Normothermic Ex Vivo Liver Platform Using Porcine Slaughterhouse Livers for Disease Modeling

Melanie Krüger [1,2,†], Alicia Ruppelt [1,3,†,*], Benjamin Kappler [1], Elke van Soest [1], Roos Anne Samsom [2], Guy C. M. Grinwis [4], Niels Geijsen [2], J. Bernd Helms [5], Marco Stijnen [1], Linda M. Kock [1,6], Marco Rasponi [3], Hans S. Kooistra [2] and Bart Spee [2]

Table S1. Primers used for DNA analysis.

Gene	Forward	Reverse
YWHAZ	CAAAGACAGCATTTGATGAAGCC	ATCTCCTTGGGTATCCGATGTC
RSP19	AAAGAAACGGTGTCATGCCC	AGGCCTTTCCCATCTTGGT
ALB	CGCTCATAGTTCGTTACACC	CTTACAACACCTAGAGCCCA
CYP3A22	CATCAACACGAAAGAAATCTTTGGG	GTCTCGTGGGTTGTTGAGG
HNF4A	CTTCTTTGACCCAGATGCC	GTCGTTGATGTAATCCTCCAG
FAH	CCAAGATGTCTTTGATCAGCCA	CCGAAGTTCTGTGTCATCTCTG
TTR	AATATGCAGAGGTTGTGTTCACAG	CTGTGGTGGAGTAAGAGTAGGG

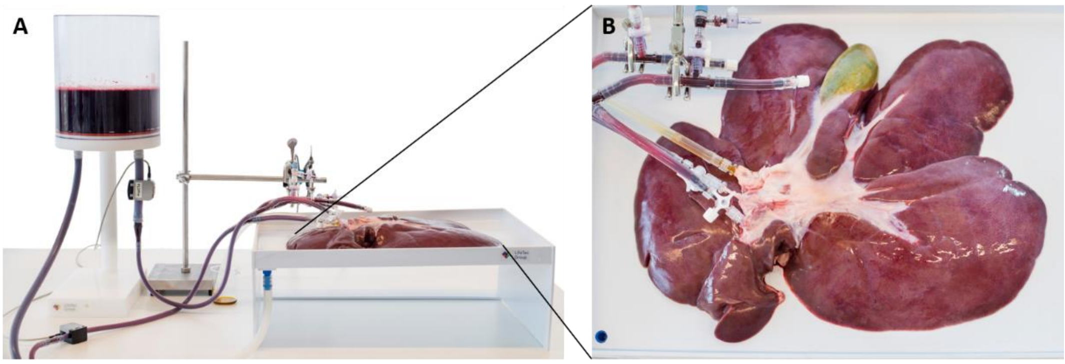

Figure S1. (**A**) Image of the custom-made liver receptacle with reservoir supplying the static portal vein pressure. (**B**) Cannulated porcine liver with gall bladder.

Table S2. Blood gas analysis, hematology, pressures and flows, bile production and liver weight for untreated at timepoints 0, 30, 60, 300 min of ex vivo perfusion as mean and SD over n = 3 livers (untreated).

	Untreated					
**Timepoint [Min]**	**0**		**60**		**300**	
Analyte	Mean	SD	Mean	SD	Mean	SD
Free fatty acids [mmol/L]	0.25	0.06	0.20	0.07	0.12	0.01
Urea [mmol/L]	2.73	0.12	3.90	0.22	8.90	0.67
AST [U/L]	89.33	56.45	2301.67	868.53	4304.00	1347.76
LDH [U/L]	796.67	12.47	1966.67	402.77	3000.00	778.89
Ammonia [µmol/L]	511.67	214.49	169.00	63.61	150.67	77.96
Lactate [mmol/L]	9.17	2.65	10.72	2.67	10.66	6.39
Portal vein pressure [mmHg]	4.19	1.32	5.50	0.16	8.49	1.28
Hepatic artery pressure [mmHg]	28.90	6.00	65.23	4.62	84.70	4.99
Portal vein Flow [mL/min]	211.33	158.77	668.67	108.30	864.33	133.18
Hepatic artery flow [mL/min]	13.67	10.66	263.67	85.17	379.67	92.78
pH	7.36	0.03	7.31	0.02	7.37	0.01

Glucose [mmol/L]	5.87	0.83	9.97	3.41	5.53	1.38
Hct [%]	27.00	2.16	25.33	1.70	25.67	2.62
Hb [g/dL]	9.20	0.73	8.60	0.59	8.73	0.88
Bile [g]			1.43	0.74	25.13	4.97
Weight	2566	245.14			2730.67	78.4

Table S3. Blood gas analysis, hematology, pressures and flows, bile production and liver weight for free fatty acid (FFA) treated livers at timepoints 0, 30, 60, and 300 min of ex vivo perfusion as mean and SD over $n = 2$ livers (FFA_bol, FFA_cts).

	**FFA_bol**						**FFA_cts**					
**Timepoint [Min]**	**0**		**60**		**300**		**0**		**60**		**300**	
Analyte	Mean	SD	Mean	SD	Mean	SD	Mean	SD	Mean	SD	Mean	SD
Free fatty acids [mmol/L]	2.48	0.16	1.24	0.04	0.22	0.02	0.10	0.02	0.28	0.01	0.20	0.02
Urea [mmol/L]	2.65	0.05	3.50	0.10	8.40	0.60						
AST [U/L]	65.00	1.00	2648.50	333.50	4339.00	634.00	6398.00	1904.00	6769.00	1481.00	7497.50	1284.50
LDH [U/L]	885.00	515.00	2500.00	300.00	3500.00	100.00	246.50	78.50	240.50	74.50		
Ammonia [μmol/L]	480.00	120.00	695.00	25.00	530.00	125.00						
Lactate [mmol/L]	6.72	1.26	9.79	1.71	22.80	5.20						
Portal vein pressure [mmHg]	3.95	0.25	5.50	0.30	7.81	0.71	9.00	0.10	9.00	0.10	9.15	0.25
Hepatic artery pressure [mmHg]	31.25	5.75	47.75	2.75	84.00	3.00	78.90	3.00	79.70	1.50	79.95	0.85
Portal vein Flow [ml/min]	175.50	94.50	545.00	43.00	700.00	6.00	572.00	39.00	591.00	39.00	560.00	28.00
Hepatic artery flow [ml/min]	95.00	0.00	101.00	37.00	302.50	38.50	505.00	23.00	541.50	11.50	548.00	10.00
pH	7.36	0.02	7.36	0.00	7.30	0.03	7.25	0.02	7.28	0.05	7.31	0.02
Glucose [mmol/L]	3.95	0.75	18.40	4.90	16.75	5.65	15.05	7.25	9.90	1.40	7.85	0.35
Hct [%]	25.50	4.50	19.00	6.00	23.00	0.00	26.00	0.00	23.50	0.50	23.00	1.00
Hb [g/dL]	8.65	1.55	6.80	1.70	7.80	0.00	8.80	0.00	8.00	0.20	7.85	0.35
Bile [g]			1.40	0.00	14.15	2.85	2.19	0.83	17.70	2.10	21.54	0.66
Weight [g]	2563.50	38.50			2939.50	100.50	2255.35	216.05			2241.00	0.00

Table S4. Hematology parameters and weight for livers treated with 155 mg/L and 300 mg/L acetaminophen (APAP) as mean and SD for $n = 3$ livers over 5 h of perfusion.

	**APAP_155**						**APAP_300**					
**Timepoints [Min]**	**0**		**60**		**300**		**0**		**60**		**300**	
Analyte	Mean	SD	Mean	SD	Mean	SD	Mean	SD	Mean	SD	Mean	SD
Glucose [mmol/L]	16.23	13.28	10.57	10.74	8.35	5.85	6.60	1.00	5.80	0.75	5.37	0.61
Urea [mmol/L]	4.03	0.49	8.43	0.92	10.50	1.41						
AST [U/L]	3208.67	827.97	5189.00	1618.25	6056.33	1800.64	5965.00	2727.25			8768.67	2830.80
ALT [U/L]	193.33	42.32	266.33	79.32	292.67	85.19	232.67	76.04			289.33	74.00
Ammonia [μmol/L]	273.67	16.03	228.00	41.79	224.33	38.47						
Lactate [mmol/L]	14.43	2.11	20.50	2.46	23.60	5.61						
Albumine [g/L]	26.67	2.87	24.67	3.30	26.00	3.27						
Portal vein pressure [mmHg]	6.20	1.98	8.80	0.99	9.93	0.38	8.53	2.05	7.10	3.00	8.23	2.27
Hepatic artery pressure [mmHg]	73.30	2.62	74.97	6.82	63.57	22.49	81.90	3.81	82.13	3.11	82.20	3.27
Portal vein Flow [mL/min]	648.67	122.50	660.00	83.22	538.33	184.92	527.33	91.23	528.00	87.55	512.67	85.99
Hepatic artery flow [mL/min]	331.67	17.75	384.00	32.78	299.00	180.73	201.77	104.98	229.93	122.25	225.00	123.26

pH	7.33	0.01	7.28	0.08	7.18	0.19	7.25	0.03	7.29	0.05	7.29	0.05
Hct [%]	21.00	2.16	19.67	1.70	19.67	2.62	25.67	3.30	24.67	3.40	24.67	3.30
Hb [g/dL]	7.17	0.74	6.70	0.59	6.67	0.91	8.70	1.13	8.37	1.14	8.40	1.13
Bile [g]	11.40	0.00	27.00	0.00	24.17	4.88	5.45	5.69	7.10	5.87	7.83	5.78
Weight [g]	2480.47	178.29			2911.17	67.13	2168.17	152.42			2579.47	379.14
